# Pediatric hepatocellular carcinoma associated with Niemann–Pick disease type C: Case report and literature review

**DOI:** 10.1002/jmd2.12344

**Published:** 2022-11-09

**Authors:** Soojin Hwang, Yunha Choi, Beom Hee Lee, Jin‐Ho Choi, Ja Hye Kim, Han‐Wook Yoo

**Affiliations:** ^1^ Department of Pediatrics, Asan Medical Center Children's Hospital University of Ulsan College of Medicine Seoul Republic of Korea; ^2^ Medical Genetics Center, Asan Medical Center Children's Hospital University of Ulsan College of Medicine Seoul Republic of Korea

**Keywords:** hepatocellular carcinoma, hypoglycemia, Niemann–Pick disease type C, NPC1

## Abstract

Niemann–Pick disease type C (NPC) is a rare, autosomal recessive, lysosomal storage disease, resulting from mutations in the cholesterol trafficking proteins NPC1 or NPC2, which is characterized by progressive neurodegeneration and hepatic dysfunction. The hepatic involvement in NPC is usually neonatal cholestasis and hepatosplenomegaly. Only a few cases of severe hepatic complications were reported including acute liver failure, cirrhosis, and hepatocellular carcinoma (HCC). We described the case of a 6‐year‐old male with NPC with HCC. He had a history of neonatal cholestasis and motor delay. At the age of 6 months, he was diagnosed with NPC, which was confirmed by the detection of a compound heterozygous *NPC1* mutation (p.C113Y/p.A927V). He presented recurrent hypoglycemia and abdominal distension. An ultrasound, computed tomography scan, and biopsy revealed that he had a stage IV HCC with pulmonary metastasis. With the literature review and this case, HCC can be a rare fatal comorbid condition in patients with NPC, particularly infantile‐onset, male patients with a relatively long disease history, necessitating appropriate HCC surveillance.

**Synopsis**

Niemann–Pick disease type C is associated with pediatric hepatocellular carcinoma, requiring new guidelines for cancer surveillance.

## 1 INTRODUCTION

Niemann–Pick disease type C (NPC) is a lysosomal lipid storage disease with autosomal recessive inheritance, which is caused by mutations in *NPC1* (95%) or *NPC2* (5%) genes.^1^ The overall incidence of NPC is approximately 1 in 100,000 to 120,000 live births.^2^ Mutations in either *NPC1* (OMIM #257220) or *NPC2* (OMIM #607626) genes encoding proteins involved in cholesterol trafficking, lead to the accumulation of unesterified cholesterol and glycolipids in the lysosome. NPC is clinically characterized by progressive neurological deterioration and hepatosplenomegaly.^3^ The age at neurological onset in NPC is critical to predicting disease course and therapeutic considerations.^4^ Clinical manifestations of NPC vary depending on the age at disease onset. During infancy, most patients present a visceral‐neurodegenerative form. Neurological symptoms and signs include developmental delay, seizure, hypotonia, spasticity, vertical supranuclear gaze palsy, and ataxia. In specific, the predominant features of visceral symptoms are hepatosplenomegaly and persistent cholestatic jaundice.^5^ In the majority of patients with NPC, liver diseases are usually spontaneously resolved, but cirrhosis and liver failure remain in some cases. Furthermore, hepatocellular carcinoma has been reported in only a few patients with NPC and fatal outcomes.^6^, ^7^, ^8^, ^9^

Hepatocellular carcinoma (HCC) is a rare type of solid cancer in children with 0.5%–1.5% of all pediatric malignancies.^10^ HCC often occurs in older adolescents and has been reported in children younger than 5 years old. A cirrhotic or noncirrhotic condition involved in viral infections, nonalcoholic fatty liver diseases, and inborn errors of metabolism like tyrosinemia type I and glycogen storage disease type I and IV increase the risks of HCC in childhood.^11^ We experienced fulminating HCC in a 6‐year‐old boy with NPC. This short report described the case and reviewed the literature to emphasize the necessity of routine HCC surveillance in clinical practice.

## 2 CASE REPORT

**FIGURE 1:**
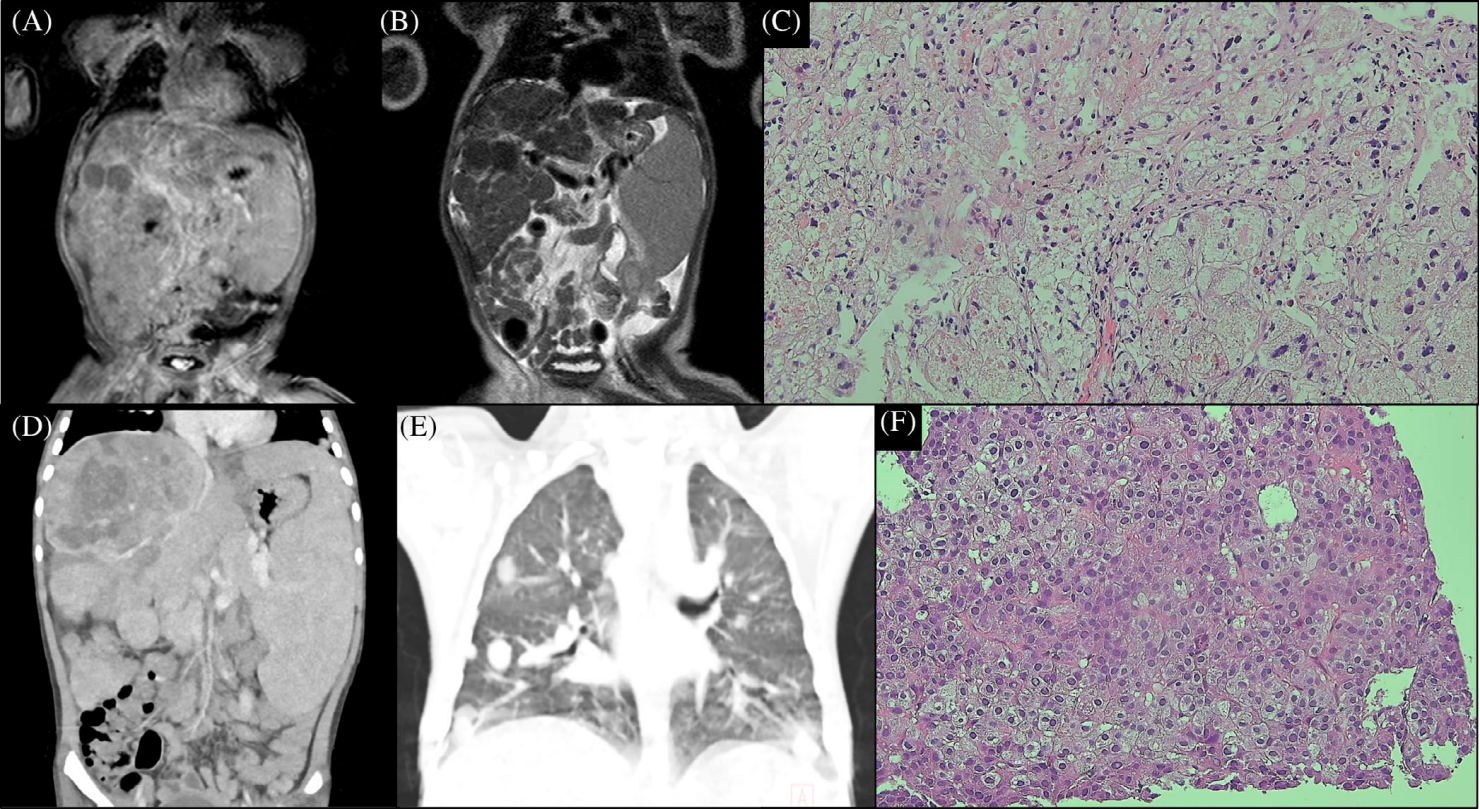
Images and liver biopsy at diagnosis of NPC (at 6 months old, A–C) and HCC (at 6 years old, D–F). (A) Coronal T1‐weighted image of MRI, (B) coronal T2‐weighted image of MRI, (C) liver biopsy showing hepatitis with lipid‐laden macrophages and perisinusoidal fibrosis (hematoxylin and eosin [H&E], ×200), (D) abdominal CT scan showing liver cirrhosis with a large hypervascular mass and splenomegaly, (E) chest CT scan showing multiple pulmonary metastases, (F) liver biopsy confirming confirms HCC of Edmondson**–**Steiner grade 2 ([H&E], ×200)

The patient was a 6‐year‐old Korean boy and the only child of nonconsanguineous healthy parents pregnant by in vitro fertilization. He was born appropriate for gestational age at full term. In addition, he was born through the elective cesarean section with normal Apgar scores and no perinatal complications. He was brought to medical attention because of fever and persistent jaundice at age of 2 months. In the physical examination, the patient was generally icteric. The liver and spleen were palpable 3 finger's breadth below the costal margin. Laboratory tests revealed anemia (Hb 8.5 g/dl); normal leukocyte and platelet counts; increased AST 214 IU/L (≤40 IU/L), ALT 95 IU/L (≤40 IU/L), γ‐GT 103 IU/L (8–61 IU/L), ALP 405 IU/L (40–120 IU/L); total bilirubin 10.0 mg/dl (0.2–1.2 mg/dl); direct bilirubin 7.9 mg/dl (≤0.5 mg/dl); PT 1.26 INR (0.8–1.3 INR); and aPTT 40.8 s (24–33 s). These laboratory tests suggest neonatal cholestatic liver disease. The total cholesterol 145 mg/dl (≤199 mg/dl), triglyceride 167 mg/dl (≤199 mg/dl), and HDL‐cholesterol 44 mg/dl (≥40 mg/dl) were normal. Tests for congenital infection, viral hepatitis, and metabolic screening were negative. Abdomen ultrasound and liver MRI showed marked hepatomegaly with multifocal nodularity and splenomegaly (Figure 1A,B). The liver biopsy demonstrated a giant cell transformation with ballooning degeneration, pinkish intracytoplasmic globules, and perisinusoidal fibrosis (Figure 1C). The whole‐exome sequencing was done and it revealed compound heterozygous missense mutations in *NPC1* (c.338G>A:p.C113Y, c.2780C>T:p.A927V), confirming the diagnosis of NPC at age of 6 months. The Filipin test was positive in his skin fibroblasts. The concentration of the biomarker lyso‐SM‐509 was increased to 1.5 ng/ml (<0.9 ng/ml).

**FIGURE 2:**
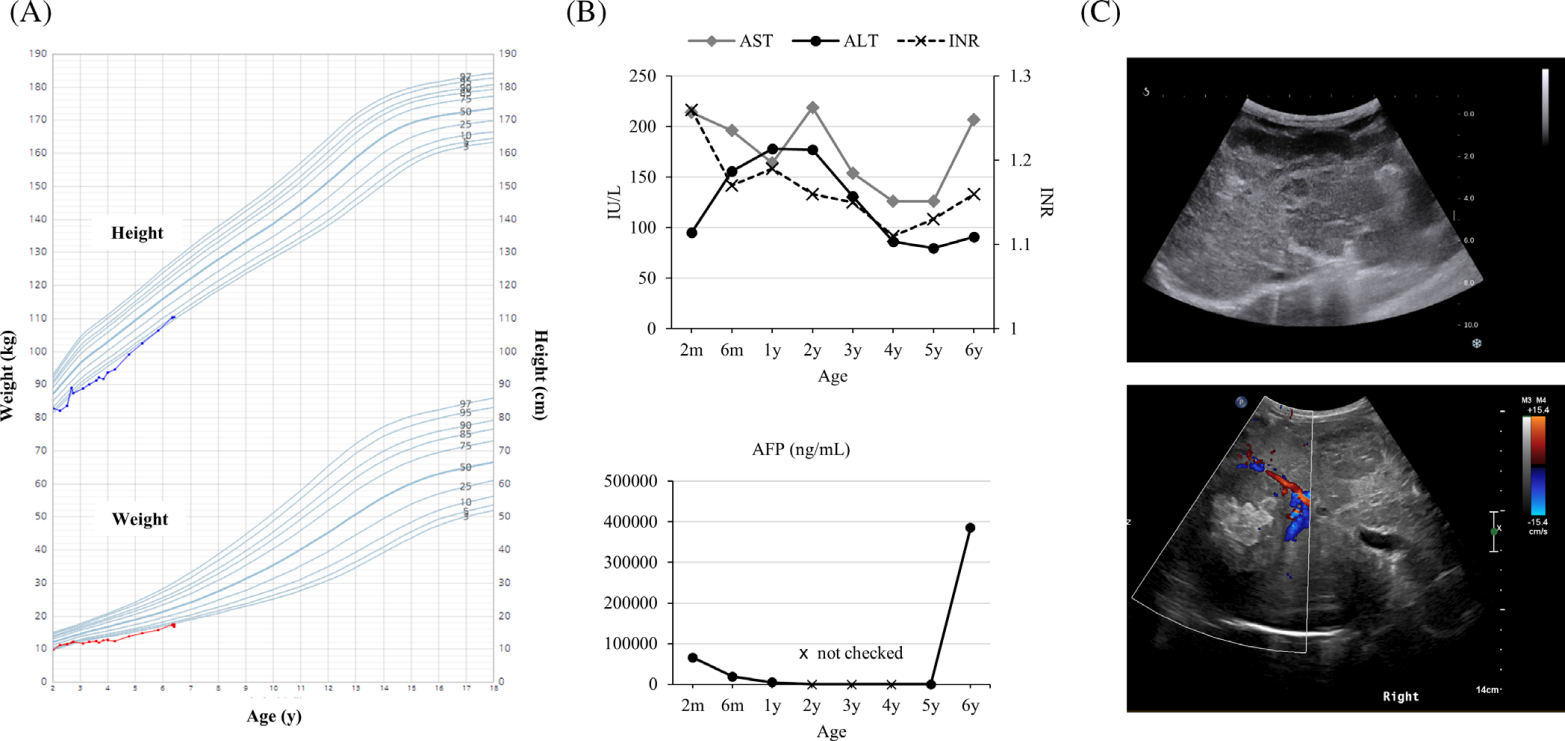
Clinical parameters and images of the patient. (A) Growth chart, (B) laboratory findings including transaminase, INR, and AFP, (C) liver ultrasound images showing cirrhotic liver with nodules at the last visit (upper panel), a large hyperechoic mass newly appeared at diagnosis of HCC (lower panel)

Since then, he continued regular clinic visits every 6 months. Physical examination, developmental assessment, and laboratory tests were performed every visit. Annual abdominal ultrasound was carried out except for the previous visit. At that time, his parents canceled the scheduled test at their request. The clinical course and parameters were described in Table 1 and Figure 2. His height and weight were in the third percentile for age (Figure 2A). Regarding developmental milestones, he was just crawling at age 1 year and showed gross and fine motor delay. Miglustat (Zavesca®, Actelion Pharmaceuticals, Allschwil, Switzerland) treatment was initiated. He could walk alone at age 3 years. Otherwise, no further neurological sign was observed until the age of 6 years with normal intelligence. Systemic symptoms including cirrhotic liver with elevated transaminases and splenomegaly were stationary for a while (Figure 2B,C upper panel). Leaving a lot to be desired, the alpha‐fetoprotein (AFP) level was not checked for the last few years (Figure 2B lower panel).

**TABLE 1:** Clinical follow‐up data of the patient

Age	Height SDS	Weight SDS	Development	Hepatomegaly (FB)	Splenomegaly (FB)	AST (IU/L)	ALT (IU/L)	PT (INR)	AFP (ng/ml)	Abdomen ultrasonography
2m	0.29	0.13	Head control	3	3	214	95	1.26	65,700	Cirrhotic multinodular liver, splenomegaly (8.6 cm)
6m	0.27	−1.39	Roll over	3	3	196	156	1.17	18,800	Cirrhotic multinodular liver, splenomegaly (7.5 cm)
1y	−1.12	−1.54	Crawling (motor delay since then)	2	2	164	178	1.19	4660	Cirrhotic multinodular liver, splenomegaly (8.2 cm)
2y	−1.38	−1.88	Stand with support	2	2	219	177	1.16	NC	Cirrhotic multinodular liver, splenomegaly (8.3 cm)
3y	−2.3	−2.39	Walk alone	2	2	154	131	1.15	NC	Cirrhotic multinodular liver, splenomegaly (8.6 cm)
4y	−2.4	−2.68	Climb stairs with hand support	2	3	126	86	1.11	NC	Cirrhotic multinodular liver, splenomegaly (10.42 cm)
5y	−1.98	−2.47	Hop on one foot	2	3	126	80	1.13	NC	NA
6y	−1.72	−2.11	Apparently normal development	4	4	192	78	1.14	385,202	A large nodule with hyperechoic foci newly appeared in cirrhotic liver, severe splenomegaly (15.1 cm). HCC was identified.

Abbreviations: FB, finger's breadth; NA, not available; NC, not checked.

At the age of 6 years, he was brought to the emergency room with dizziness, sweating, and lethargy. Physical examination demonstrated enlarged liver in hard consistency, palpable 4 finger's breadth below the costal margin with the palpable spleen. The laboratory tests revealed severe hypoglycemia (serum glucose 28 mg/dl) and increased AST 192 IU/L, ALT 78 IU/L, ALP 206 IU/L, and PT 1.14 INR. An intravenous glucose infusion with a 10% dextrose was done and serum glucose level was raised to 144 mg/dl. He revisited the emergency room with the same symptoms on the following day. Serum glucose level was 27 mg/dl. Further laboratory tests at critical samples revealed low insulin 0.6 μIU/ml (0.9–8.5 μIU/ml), C‐peptide 0.10 μIU/ml (0.48–3.3 ng/ml), and β‐hydroxybutyrate 0.2 mmol/L (≤0.6 mmol/L), excluding hyperinsulinism. Serum growth hormone, cortisol, and thyroid hormone levels were normal. An abdominal ultrasound showed multiple hyperechoic nodules in the liver and splenomegaly (Figure 2C lower panel). Additional liver dynamic computed tomography (CT) demonstrated cirrhosis and there were multiple malignant hepatic tumors with a large vascularized mass of 10 cm diameter in the right liver (Figure 1D). A chest CT showed multiple lung metastases (Figure 1E). The liver biopsy confirmed the diagnosis of HCC (Figure 1F). AFP level was elevated to 385,202 ng/ml (Figure 2B lower panel). Prothrombin induced by vitamin K absence or antagonist‐II (PIVKA‐II) level was also increased to 156 mAU/ml (<40 mAU/ml), strongly suggesting HCC. Due to the advanced stage of HCC, palliative therapy was available. His parents did not want chemotherapy and surgery and considered hospice care. After being hospitalized for 2 weeks, he was discharged home with total parenteral nutrition and home visiting nursing care.

## 3 DISCUSSION

To date, four NPC cases with HCC have been in the literature.^6^, ^7^, ^8^, ^9^ Table 2 summarizes the clinical findings of five patients with NPC and HCC including the present case. Males (80%) are more predominantly affected than females. In general, HCC itself affects more males than females (male to female ratio; 3:1).^10^ Three patients (60%) had neurologic deficits. Our patient showed gross and fine motor delay at age 1 year, who was an early‐infantile NPC. Two patients in the literature were late‐infantile NPC; the age of neurological onset was 3.5 years in Gartner et al.,^6^ and 2 years in Rodriguez‐Gil et al.^9^ However, the patient in Birch et al.^8^ showed normal development and Pennington et al.^7^ did not describe neurological symptoms. All reported patients with NPC and HCC manifested visceral‐neurodegenerative symptoms in the infancy. Symptoms started at around 2 months and the diagnosis of NPC was usually established at around 2 years. Their clinical presentations and history are not particularly different from other patients with NPC. All described patients typically presented neonatal cholestatic hepatitis and some had neurological symptoms later. Visceral symptoms including hepatomegaly, splenomegaly, cholestasis, and hepatitis prevailed before the onset of neurodegenerative symptoms. The median age at diagnosis of HCC was 6 years old, which ranged from 4 to 12 years old. Most patients were diagnosed at advanced stage III or IV, resulting in poor prognosis.

**TABLE 2:** Clinical characteristics of reported patients with hepatocellular carcinoma in Niemann–Pick disease type C^a^

Reference	Type	Gender	Age at diagnosis	Clinical symptoms	Medication	Age at HCC diagnosis	Hepatic presentation	HCC stage	Clinical course
Gartner et al., 1986^6^	Not known (C)^b^	Female	Not known (2 months‐6.5 years)	Hepatosplenomegaly, mild cholestatic hepatitis, spasticity, ataxia, dysarthria, supranuclear gaze palsy, seizure, swallowing difficulty, and intellectual disability	None	6.5 years	Incidental HCC was founded by liver biopsy	Not known	Liver transplantation. Progressive intellectual and neurological deterioration.
Pennington et al., 1996^7^	C	Male	5 months	Hepatosplenomegaly, biliary cirrhosis, and others were not reported	None	4 years	Nausea, vomiting, diarrhea, abdomen distension with ascites	IV (pulmonary metastasis)	Uncontrolled esophageal variceal bleeding led to shock and death.
Birch et al., 2003^8^	C	Male	2.5 years	Mild cholestatic hepatitis, hepatosplenomegaly, and normal development	None	6 years	Abdominal pain and distension with ascites, portal vein obstruction, fever (AFP 400724 ng/ml)	IV (pulmonary metastasis)	Comfort care. Death.
Rodriguez‐Gil et al., 2021^9^	C1 (p.G1146V and p.L1248Vfs*3 of NPC1)	Male	2 years	Cholestatic hepatitis, hepatosplenomegaly, developmental delay, cerebellar symptoms, and cognitive decline	Miglustat since 2 years, hydroxypropyl β‐cyclodextrin since 6 years	12 years	Abdominal pain, ascites, portal vein thrombosis, fever (AFP 16155 ng/ml)	III (intrahepatic metastasis)	Hepatic lobectomy and adjuvant chemotherapy. Recurrence with pulmonary metastasis led to death.
Hwang et al.^c^	C1 (p.C113Y and p.A927V of NPC1)	Male	6 months	Cholestatic hepatitis, hepatosplenomegaly, liver cirrhosis, motor delay, normal intelligence	Miglustat since 1 year.	6 years	Recurrent hypoglycemia, abdominal distension, fever (AFP 385202 ng/ml)	IV (pulmonary metastasis)	Comfort care.

^a^ Case reports identified by PubMed search with keywords “Niemann–Pick disease” and “Hepatocellular carcinoma.”

^b^ Sphingomyelinase activity was normal.

^c^ Present report.

Two of five patients were treated with miglustat, and one patient concomitantly took 2‐hydroxypropyl‐β‐cyclodextrin (HPβCD). The therapeutic options for NPC are still limited. Miglustat is the only approved drug for NPC in Europe.^12^ This iminosugar inhibits glucosylceramide synthase and is originally developed for treating Gaucher disease, known to stabilize neurological symptoms of NPC. Furthermore, recent research has demonstrated that the cyclic oligosaccharide HPβCD drives the redistribution of cholesterol derivatives and may reduce the neurodegenerative process in NPC.^13^ However, the long‐term effects of miglustat, HPβCD, and combination therapy on NPC are not clear. Our patient started miglustat therapy at age 1 year because he showed gross and fine motor delay. Later, he has caught up with his delayed developmental milestones with normal intelligence. He did not present any abnormal neurological signs, but hepatomegaly and abnormal liver function persisted. The effects of miglustat on organomegaly and liver functions remained unclear. It seems to have little effect.^14^

Hepatic involvement in metabolic disease is common and critical since the liver has a pivotal role in various metabolic pathways. Liver cancers such as HCC and hepatic adenoma are often accompanied by some inborn errors of metabolism.^15^ The first report that a patient with NPC developed HCC was described in 1986.^6^ It is noted that, as shown in Table 2, a total of five patients were reported and HCC, a rare form of cancer in children, occurred at around 6 years old. Interestingly, there is a time lag between the age at diagnosis of NPC and the age at diagnosis of HCC. It takes about 3.5 years to 10 years. Initial hepatic presentations of HCC were commonly abdominal distension and abdominal pain. The patient in this study presented recurrent severe hypoglycemia. Although hypoglycemia is an uncommon sign of HCC, in which cancer must be considered in the differential diagnosis. Hypoglycemia can occur either by the glucose consumption of tumor or insulin‐like growth factor‐2 (IGF‐2) production as paraneoplastic complications.^16^ Unfortunately, the IGF‐2 level was not measured in this case.

Although the underlying pathophysiologic mechanism of HCC in NPC is still unclear, cirrhosis is a prerequisite of HCC, which is a malignant epithelial tumor. Toxic metabolites like unesterified cholesterol in NPC are potentially responsible for developing HCC and they induce oxidative stress and chronic inflammation, which causes chronic hepatic injury and fibrosis.^17^, ^18^ In addition, a previous study demonstrated that NPC2 expression decreased in human HCC, and downregulation of NPC2 promoted tumorigenesis via MAPK/ERK pathways. Moreover, the reduced expression of NPC2 and Niemann–Pick C1‐Like 1 (NPC1L1), a homolog of NPC1 protein was observed in HCC, indicating poor prognostic factors in HCC patients.^19^ To date, most patients with NPC and HCC are reported to carry biallelic mutations in the *NPC1* gene.

Based on the literature review of five NPC cases with HCC including this case, patients with NPC are at increased risk of HCC in childhood. Overall, we suggest that infantile‐onset, male patients with NPC and relatively long disease histories are particular risk factors for the development of HCC. Miglustat therapy itself does not seem to be a risk factor. However, it may modify the natural history of NPC by improving the survival time of infantile‐onset patients with NPC. Once HCC is diagnosed, it often ends up fatal outcome because of the delayed diagnosis and rapid progression. Recent clinical guidelines and management for NPC were published in 2018.^20^ It does not address this issue. AFP and ultrasound monitoring may be suitable for detecting HCC. In our case, annual abdominal ultrasound was performed, however, unfortunately AFP monitoring was not continued. Our experience suggested that surveillance for HCC using AFP and ultrasound monitoring at least on an annual basis should be mandatory in patients with infantile‐onset NPC.

In conclusion, HCC can be a rare fatal comorbid condition in patients with NPC, particularly infantile‐onset, male patients with relatively long disease history, necessitating appropriate HCC surveillance.

## CONFLICT OF INTEREST

The authors declare no conflicts of interest.

## ETHICS STATEMENT

All procedures were followed by the ethical standards of the responsible committee on human experimentation and with the Helsinki Declaration of 1975, as revised in 2000. This study was approved by the institutional review board (IRB) of the Asan Medical Center, Seoul, Korea (IRB No. 2022‐0230).

## INFORMED CONSENT

Written informed consent was obtained directly from the parents.

## INSTITUTIONAL COMMITTEE FOR CARE AND USE OF LABORATORY ANIMALS

This article does not contain any studies with animal subjects.

## Data Availability

Data are available on request from authors.
